# The Effective Strategies to Avoid Medication Errors and Improving Reporting Systems

**DOI:** 10.3390/medicines8090046

**Published:** 2021-08-27

**Authors:** Abbas Al Mutair, Saad Alhumaid, Abbas Shamsan, Abdul Rehman Zia Zaidi, Mohammed Al Mohaini, Alya Al Mutairi, Ali A. Rabaan, Mansour Awad, Awad Al-Omari

**Affiliations:** 1 Research Center, Almoosa Specialist Hospital, Al-Ahsa 36342, Saudi Arabia; 2 College of Nursing, Princess Norah Bint Abdulrahman University, Riyadh 12214, Saudi Arabia; 3 School of Nursing, University of Wollongong, Wollongong, NSW 2522, Australia; 4 Administration of Pharmaceutical Care, Al-Ahsa Health Cluster, Ministry of Health, Al-Ahsa 36342, Saudi Arabia; saalhumaid@moh.gov.sa; 5 Research Center, Dr. Sulaiman Al Habib Medical Group, Riyadh 12214, Saudi Arabia; abbas.shamsan@drsulaimanalhabib.com (A.S.); ar-zia@hotmail.com (A.R.Z.Z.); research.center@drsulaimanalhabib.com (A.A.-O.); 6 College of Medicine, Alfaisal University, Riyadh 12214, Saudi Arabia; 7 Basic Sciences Department, College of Applied Medical Sciences, King Saud Bin Abdulaziz University for Health Sciences, Alhasa 31982, Saudi Arabia; maam670@gmail.com; 8 Basic Sciences Department, College of applied Medical Sciences, King Abdullah International Medical Research Center, Alhasa 31982, Saudi Arabia; 9 Department of Mathematics, Faculty of Science, Taibah University, Medina 54321, Saudi Arabia; amutairi@taibahu.edu.sa; 10 Microbiology Department, Johns Hopkins Aramco Healthcare, Alhasa 31982, Saudi Arabia; arabaan@gmail.com; 11 Department of Public Health and Nutrition, The University of Haipur, Haripur 22610, Pakistan; 12 Commitment Administration, General Directorate of Health Affairs, Ministry of Health, Medina 54321, Saudi Arabia; Maaalmutairi2@moh.gov.sa

**Keywords:** medical errors, medication error, improve, medication error reporting program, health care professional, patients, health organizations

## Abstract

Background: Population-based studies from several countries have constantly shown excessively high rates of medication errors and avoidable deaths. An efficient medication error reporting system is the backbone of reliable practice and a measure of progress towards achieving safety. Improvement efforts and system changes of medication error reporting systems should be targeted towards reductions in the likelihood of injury to future patients. However, the aim of this review is to provide a summary of medication errors reporting culture, incidence reporting systems, creating effective reporting methods, analysis of medication error reports, and recommendations to improve medication errors reporting systems. Methods: Electronic databases (PubMed, Ovid, EBSCOhost, EMBASE, and ProQuest) were examined from 1 January 1998 to 30 June 2020. 180 articles were found and 60 papers were ultimately included in the review. Data were mined by two reviewers and verified by two other reviewers. The search yielded 684 articles, which were then reduced to 60 after the deletion of duplicates via vetting of titles, abstracts, and full-text papers. Results: Studies were principally from the United States of America and the United Kingdom. Limited studies were from Canada, Australia, New Zealand, Korea, Japan, Greece, France, Saudi Arabia, and Egypt. Detection, measurement, and analysis of medication errors require an active rather than a passive approach. Efforts are needed to encourage medication error reporting, including involving staff in opportunities for improvement and the determination of root cause(s). The National Coordinating Council for Medication Error Reporting and Prevention taxonomy is a classification system to describe and analyze the details around individual medication error events. Conclusion: A successful medication error reporting program should be safe for the reporter, result in constructive and useful recommendations and effective changes while being inclusive of everyone and supported with required resources. Health organizations need to adopt an effectual reporting environment for the medication use process in order to advance into a sounder practice.

## 1. Introduction

Medical errors are described as unintentional mistakes either by omission or commission. Medical errors are classified into an error of execution or an error of planning, which are explained as the unsuccessful process of deliberate action or utilization of an improper plan to attain a goal, respectively, or by deviating from the process of care that may potentially cause harm to the patient [1]. In 2008, the US Department of Health and Human Services Office reported 180,000 deaths by medical errors among hospitalized patients [1]. A high percentage of medical errors is attributed to medications that account for almost 1.5 million victims of medical errors every year [2]. The National Coordinating Council for Medication Error Reporting and Prevention (NCCMERP) defines a medication error as “any preventable event that may cause or lead to inappropriate medication use or patient harm while the medication is in the control of the health care professional, patient, or consumer.” These events can be linked to procedures, healthcare commodities, professional practice, along with systems consisting of prescription, order communication, dispensing, monitoring, product labeling, distribution, compounding, administration, nomenclature and packaging, education, and use. These events can be linked to healthcare commodities, procedures, professional practice, along with systems started with nomenclature and packaging, storing and distributing, prescribing, transcribing, documenting, reviewing, preparing (or compounding), product labeling, educating, dispensing, and ended with drug administration and monitoring [3]. Medication errors significantly impact the well-being of individuals, organizations, and healthcare systems. According to an NCCMERP report, medication errors are ranked the sixth cause of mortality in the United States, with 5–10% of the reported medication errors classified as harmful [3]. Recently, medication errors have become a challenge facing healthcare systems and are directly linked to hospital mortality and morbidity rates [4]. Specifically, medication errors cause adverse effects on hospitalized patients and weaken the public’s confidence in the healthcare system and the healthcare services being provided [5]. In addition, medication errors negatively impact clinical outcomes such as length of stay (LOS), incurring substantial costs of about USD 2000-2500 per patient [2,6]. Another issue is the high proportion of underreporting of medication errors (estimated to be 50–60%) across healthcare organizations that is attributed to the lack of medical recording systems in many hospitals [2]. Therefore, different prevention programs were implemented to monitor errors targeting triggers and/or influencing factors of medication errors [7,8,9,10] through using carefully formulated establishment-wide reporting systems to find the likely sources of medication errors [11]. Although the reporting of medication errors offers usable data for identifying areas of improvement with regard to patient safety, the advancement of patient safety is impeded and the lack of formal reporting is well recognized [12]. A variety of standards at the institutional level and a higher level of government exist for designing an effective medication error reporting system [12]. Simultaneously, the transformation of medication error reporting systems is required to facilitate easily preventable mistakes and their often-severe aftereffects [12]. Thus, understanding what hinders reporting could eventually result in superior patient care [12]. Whilst plentiful reports have studied the contributing factors [7,8,9,10], rates of prescription errors, and adverse events [13,14,15], insufficient researches have analyzed the characteristics of successful medication error reporting systems.

## 2. Material and Methods

### 2.1. Aims and Objectives

In order to give basic details about the medication error reporting culture, incidence reporting systems, effective reporting method(s), analysis of medication error reports, and also suggest recommendations to improve medication errors reporting systems, we conducted a review of currently available literature evidence.

### 2.2. Search Strategy

A total number of five electronic databases (PubMed, Ovid, EBSCOhost, Embase.com, and ProQuest) were methodically searched for articles using components derived from the subsequent subject headings and keywords: characteristics, effective, error, improve, medication, report, reporting, successful, system. Furthermore, we searched citations from relevant papers to select additional studies. The search remained limited to English language journals published between January 1998 and June 2020.

### 2.3. Inclusion and Exclusion Criteria

Readily accessible peer-reviewed, full-text articles in the English language, primary research publications of any design (quantitative and qualitative studies: observational cohort or case-control studies, clinical trials, cross-sectional and systematic reviews) were included. We looked for studies that reported medication error reporting culture, incident reporting systems, creation of effective reporting methods, analysis of medication error reports, and recommendations to enhance medication error reporting systems. The studies identified in the search were manually evaluated for applicability in this article. We also included limited articles that concentrated on medical—not medication errors and nursing practice errors. We eliminated conference papers, editorials, letters to the editor, organizational reports, opinion papers, and case reports.

### 2.4. Data Extraction and Analysis

Two reviewers (AA and SA) individually vetted titles with abstracts followed by a full article review, where any doubt remained. Disagreements between two reviewers after full-text vetting were resolved via unanimity by a third reviewer (AS) and a fourth reviewer (ARZ). The data extraction involved evidence in each relevant selected article on medication error reporting systems, reporting culture, creating an effective reporting method, analysis of medication error reports, and/or recommendations to improve medication errors reporting systems. To examine the literature, a narrative synthesis was performed due to the variety of instruments and reported data. A narrative synthesis is characterized by the textual methodology that delivers a trustworthy tale of the findings from the selected literature [16]. Additionally eligible studies were appraised using critical appraisal tools. The appraisal consists of 10 items that assess the methodological quality of a study and determines the extent to which a study has addressed the possibility of bias in its design, conduct, and analysis. The results of the appraisal have been taken into full account and used to inform the synthesis and interpretation of the results of the recommendations.

## 3. Results and Discussion

**Figure 1:**
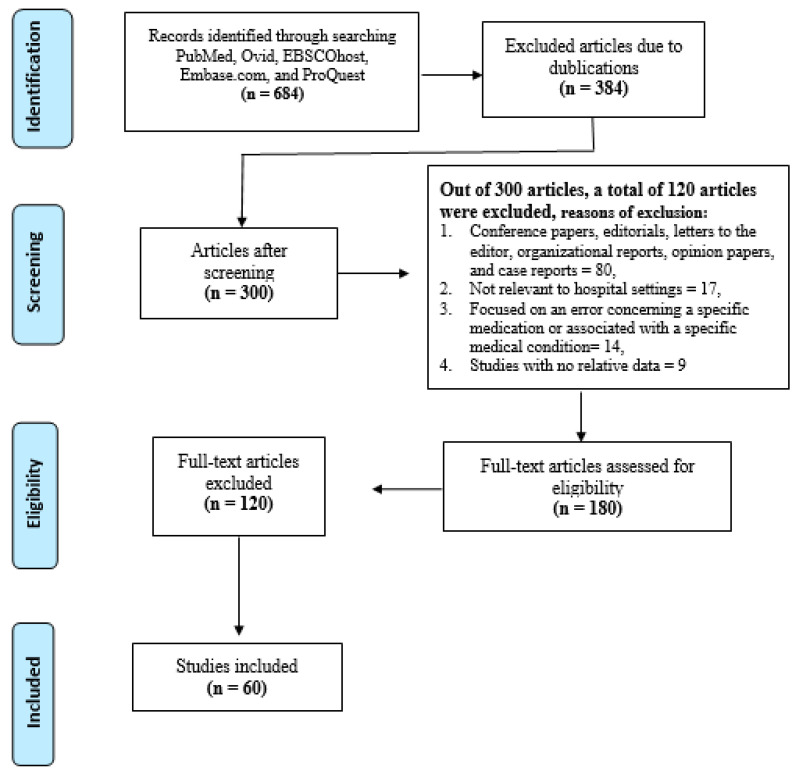
Flowchart.

Overall, we screened 5 literature databases and identified 684 articles. A total of 384 duplicated articles were excluded from the review. Then, 300 articles evaluated for possible inclusion using title and abstract. 180 articles were selected for full-text vetting, resulting in the 60 articles comprising the narrative review (Figure 1). An estimated 120 articles were omitted after full-text screening (reasons: conference papers, editorials, letters to the editor, organizational reports, opinion papers, and case reports = 80, not relevant to hospital settings = 17, focused on an error concerning a specific medication or associated with a specific medical condition = 14, or study with no relative data = 9). Articles were published from 1998 to 2020 with a summit of papers between 2006 and 2014. Articles largely came from the United States and the United Kingdom, with fewer studies from Canada, Australia, New Zealand, Korea, Japan, Greece, France, Saudi Arabia, and Egypt. 

### 3.1. Reporting Culture

A system for reporting medical errors can lead to future detection of the possibility of a medical error occurring [17,18]. However, patient safety is not developing fast enough to face future challenges in healthcare [19]. In the past, medical errors were rarely disclosed; nowadays, however, failing to disclose an error in the hospital is considered a violation of the code of ethics and leads to litigation [17,20]. Nevertheless, do all healthcare providers divulge medical errors? The decision of disclosing a medical error by a healthcare provider is problematic [17]. Fein and others discussed the most effective factors that influence decisions on disclosing a medical error, which fall into four categories; provider elements, patient elements, error elements, and institutional culture [17,18,19,20,21]. There is an absence of reporting medical errors in the medical field and factors influencing motivation to report medical errors have been investigated in several countries. Around 16–20% of nurses fail to report incidences [22,23,24,25] because they fear being terminated by employers. Some healthcare providers fail to report an incident because of a lack of management feedback [22,25,26], unsupportive colleagues [26], lack of time [25], and lack of knowledge [27]. In order to realize the development in such an area, cultural changes have to be made; feeling safe to report a medical error and learning from past mistakes are crucial factors that might improve patient safety [19,28]. One of the controversial problems in reporting systems is whether reports should be mandatory or voluntary. Mandatory reports might lead to litigations [29] and may destroy the doctor-patient relationship, which can lead health care providers to practice “defensive medicine” [29,30]. Ethically and professionally, healthcare providers should not be obligated to report medical errors. Voluntary reporting is beneficial for medical learning and promotes a culture of safety. On the other hand, mandatory reports have shown the effectiveness of participation in reporting medical errors. For example, in Denmark the reporting rate is 50% compared to 1% in Australia, where the reporting is voluntary [19]. England has changed its policy of reporting from voluntary to mandatory, and if there is a failure to notify the error, the medical Trust may face the consequence of a £4000 penalty. To have organizational accountability and to improve patients’ safety and effective prevention systems, the two reports “To Err is Human” and “An Organization with a Memory” both suggested the utilization of a compulsory reporting system in harmful accidents [19,30].

### 3.2. Incidence Reporting Systems

Incidence Reporting Systems (IRSs) have been known to minimize incidences in air flights; hypothetically, it would also decrease the medical errors in the healthcare systems [31]. Nowadays, medical error reporting systems are widely used. The New Zealand Pharmacovigilance Centre (NZPhvC) is the national center responsible for monitoring adverse reactions to medications in New Zealand, through the Centre for Adverse Reactions Monitoring (CARM) [32]. In Australia, the Advanced Incident Monitoring System (AIMS) was implemented around 2005 [31], and the National Reporting and Learning System (NRLS) is used since 2003 in the United Kingdom [31]. Additionally, in Ireland, the National Adverse Event Management System (NAEMS) (formally known as STARS web IRS) was implemented and has been in use since 2004 [31]. Several years back in the United States, the Medical Event Reporting System for Transfusion Medicine (MERS-TM) and United States Pharmacopeia’s MEDMARX Reporting System were introduced. The different systems the United States has launched can be represented as a high level of knowledge in reporting systems [33,34]. There are two kinds of reporting systems, voluntary and mandatory. The most significant systems are designed after the Aviation Safety Report System (ASRS) which is run by NASA for the Federal Aviation Administration; the system is voluntary and anonymous [35]. Several voluntary systems are being modeled after the Aviation Safety Report System (ASRS) such as, the Veterans Administration Patient Safety Reporting System (PSRS) [36], the Institute for Safe Medical Practice (ISMP) which is designed for medical error reporting [37], and Data Watch which is established by the United States Food and Drug Administration (US FDA) for documenting of contrary occasions stemming from medicines and therapeutic devices [38]. The Canadian Medication Incident Reporting and Prevention System (CMIRPS), which is involved in nationwide preventable medication error occurrences and reporting, was established by Health Canada, ISMP Canada, and the Canadian Institute for Health Information (CIHI) [39]. Furthermore, in Egypt, neonatal intensive care units (NICUs) utilize the Egyptian Neonatal Safety Training Network (ENSTN), which can be used confidentially and anonymously to report medical errors [40]. In Saudi Arabia, the National Pharmacovigilance Center (NPC) was established by the Saudi Food and Drug Authority (SFDA) to monitor for surveillance of the safety matters of medications and it plays a vital role in the identification of adverse drug reactions (ADRs), their evaluation and prevention [41]. Many countries such as Greece [42], Korea [43], Japan [44], and France [45] have adopted similar systems which have shown substantial positive benefits [46,47,48,49].

### 3.3. Creating an Effective Reporting Method

Creating an effective multiple-phase reporting method to lower medication errors can act to identify the baseline rates of prescription errors. Hence, this can enable a recognition of the major types of medication errors and thereby assist in risk-reduction through the application of various preventive measures [50]. A successful strategy to prevent and detect drug-related problems may involve three stages: pre-intervention phase, intervention phase, and post-intervention phase [51]. The pre-intervention phase reinforces voluntary medication error reporting in the healthcare facility by healthcare professionals utilizing standardized forms. Reports must be continuously monitored, reviewed, and documented on a daily basis throughout the pre-intervention phase [51]. During the pre-intervention phase, medication handling stages are monitored, patient records will be reviewed, and all procedures will be documented. The incident(s) and types of medication error(s) within the healthcare facility will be identified. Quantitative and qualitative analyses of the collected reports should be carried out during the intervention phase [50,51]. Multiple quantitative and qualitative data analyses can be applied here based on the data available, such as quantitative root-cause analysis or qualitative content analysis. Root factors that contribute to prescription errors that have caused or have had the possibility to cause harm “near miss” to the patient can thus be realized [50]. The intervention phase is an integral corrective phase as it should consist of training programs for the targeted healthcare providers [51]. Training programs should be directed towards the identification of medication errors, causation, the harm inflicted, and the importance of effective communication to promote patient safety parameters within the healthcare facility. The post-intervention phase ought to embrace continuous monitoring after the intervention corrective phase [51]. It should also emphasize the re-collecting of data and comparing it with the pre-intervention data. This phase studies the adherence of staff to voluntarily report the incidents of medication errors. The incident is then reported nationally through the organization’s system or online electronic-form.

## 4. Analysis of Medication Error Reports

NCCMERP has developed a medication error taxonomy tool to aid healthcare workers and organizations characterize, trace, and analyze medication errors in a standardized, methodical approach [52]. The taxonomy is useful for developing a medication error database and designing an error reporting or data collection form. Healthcare organizations should build systems and procedures to accumulate ample information required to inspect and report medication errors at the time the events occur (ideally, all the elements identified in the taxonomy). One key component of the taxonomy, which categorizes an error in accordance with the severity of the outcome on a scale from A to I, is the NCCMERP medication index [52]. Factors such as whether the error got to the patient and if the patient was affected by the error and to what level, are considered by the index. The use of the NCCMERP medication error-index is encouraged in all healthcare delivery settings [52].

## 5. Recommendations to Improve Medication Errors Reporting Systems

Every medical institution should aim towards implementing methodologies whereby patients are not put at risk due to medication errors. Healthcare organizations should proactively eliminate these by investigating errors that have both occurred and those that may potentially occur. This way, it is possible to identify methods by which the consumption of medicines is incorrectly reported and thus mitigating the health risks patients are exposed to. A consistent organizational framework is needed to monitor and measure medication safety. Encouraging reporting, monitoring, and open discussion of medication errors is key in establishing a culture of safety. The system will improve with more data entries; these can be from existing errors already known, ones that may have been missed earlier, and even other miscellaneous errors. The following (Table 1) depicts a list of necessary factors that should be considered based on the findings explored by other academics [53,54,55,56,57,58,59,60,61,62,63].

### 5.1. Blame-Free or Non-Punitive Culture

A system that can properly evaluate and rectify errors needs to be non-punitive if is to provide meaningful, applicable data [53]. There should be a system where blame is not assigned to those experiencing the errors or those that annotate them. Priorities of an effective medication error reporting system need to target pre-emptive and retroactive actions as opposed to placing blame on an individual. Corrective actions can prevent an incident recurrence, mitigate prescription errors, and enhance the long-term well-being of patients, thus improving their quality of care [54].

### 5.2. Anonymity

The reporting system should also consider maintaining anonymity in the reporting incident data, allowing the reporter to remain anonymous while reporting the medication error [54]. A lesson can be learned from Australian and British work on “open disclosure” and “being open”; this will help individuals to enhance their understanding as the majority of these are unintended and can later be seen with transparency [55].

### 5.3. Responsive and Productive

A responsive medication error reporting system stimulates internal reporting within a health organization significantly [56]. Analysis of these reports needs to be undertaken urgently, especially those that are found to be at a more critical or detrimental level; these reports, in turn, need to be made readily available to those that can take appropriate action. The response should be visible, useful, and constructive for the health care system change [56].

### 5.4. Encourage Involvement

Patient safety is the responsibility of everyone in the healthcare organization. Engaging key stakeholders will increase the acceptance of the priorities and result in the successful implementation of improvement efforts [57]. Key stakeholders can include the patient safety officer, chief executive officer, chief nursing officer, chief operating officer, chief medical officer, director of pharmacy or chief pharmacy officer, and the Pharmacy and Therapeutics (P&T) Committee chair. Thus, it can be seen that including patient education in as many programs as possible (both medical and non-medical) is of the utmost importance [57].

### 5.5. Accountability

Coordinating with senior leadership is needed to develop formal or informal authority to ensure that any unsafe practices are evaluated and immediately addressed if necessary [57]. Developing a mechanism for holding others accountable through committees or senior leaders is essential to the success of medication safety efforts [57]. Through proper education and subsequent guidance, patients themselves will be trained to prevent such medication errors and aid both the personnel and the system that is designed to help them [57].

### 5.6. Create an Environment That Supports Reporting

With the advent of modern technologies and infrastructure, it is imperative to utilize such data analyses to further attenuate medication errors. This is more possible now than ever; especially in the way that computerized physician entries tie in with the barcoded distribution of medication and conciliate one another [58]. Hospitals that utilize mechanics such as aided journal entries and an appropriate system helping them make decisions have been shown to alleviate complications and mortality rates and consequently reduce operating expenditure [59,60]. An organizational reporting system should be made user-friendly and accessible to all employees, students, and teaching staff (if not employees) [58]. System design changes should be considered to make it easy and meaningful to report; for example, minimize the number of screens or paper pages required for reporting, balance the need for detail with ease of use, and utilize check-boxes or drop-downs [59]. These methodologies will be most effective when every user is well-versed in the running and systemic architecture of the system [59].

### 5.7. Review Summary on a Regular Basis

When working to enhance a medication error reporting program, the focus should be on increasing the reporting and analysis of reports that did not result in patient harm, with the goal of decreasing harmful events [60]. Excessive focus on trends and ‘the numbers’ through monthly statistical reports can be counterproductive if it results in a de-emphasis on the analysis of root causes that can lead to corrective actions and process improvement [60]. However, a review of summary information on a quarterly, semi-annual, or annual basis is often helpful to refocus safety improvement efforts as well as identify areas of the organization that are underreporting [61].

### 5.8. System-Oriented

To fully enhance the system and keep it in a state of improvement, it is essential that individuals feel that they are not being held responsible. They should feel empowered to improve the different facets of the system [61]. Doing so will create culture of safety to be accommodated at an individual level [61]. This will also reinforce the concept that despite an error occurring due to human individual error, it would be replicable at some point due to the deficiencies present in the reporting system [61].

### 5.9. Expertise

There needs to be experts in place that can properly assess the clinical requirements of an individual case and the fundamental system architecture that allowed this to exist in the first place [50]. Such a job requires technically-aligned experts if a reporting system is to be fully utilized [50].

### 5.10. Psychological Safety

Psychological safety should be made a requirement of healthcare organizations. Essentially it is “being able to show and employ one’s self without fear of negative consequences of self-image, status, or career” [62]. Implementing these core values allows the workplace to be one where there is both trust and respect afforded to those who are part of it [62]. Doing so allows the whole mechanism of reporting systems, in its giving and receiving feedback and identification of errors, to be further enriched [62].

### 5.11. Enough Resources

The implementation of reporting systems without adequate resources will not be useful [63]. The analysis and understanding of the root/core reasons of why various errors are occurring are paramount and need an appropriate level of due-diligence afforded; such improvements may rely on fine margins and thus need attention [63].

### 5.12. Physical Wellbeing

Healthcare providers need to have good concentration and physical wellbeing, particularly in an emergency situation [64]. Deterioration of healthcare providers’ awareness or memory coordination may impact their performance and result to mediation prescription and administration errors [65]. Previously published research has revealed that sleep deprivation among healthcare providers is linked with medical errors occurrence [66]. There is an evidence that night-shift healthcare workers commit medical errors more often than their dayshift counterparts as they experience poorer quality and shorter duration of sleep [67]. Therefore, offering shorter periods of time on a night-shift and less working hours may lead to better sleep quality and less occurrences of less medication errors.

#### Limitations

As with any review, this one has some limitations. The review mainly focused on the various reporting systems and recommendations to improve medication error reporting systems. Due to the wide-net this encompasses, a narrative approach was preferentially adopted over a more systematic literature search. This preference was favored as it allowed the inclusion of evidence; conversely, this meant there was the possibility of a bias arising when selecting the different studies, and we were not able to evaluate the strength of the evidence reported. The literature present on this topic is vast and as such, it is our recommendation to further explore this topic academically to gain a more informed understanding of the various topics discussed within this report. Thus, these medication errors along with the systems in place that allow them to propagate can be further explored, giving an informed, better understood wide-scale picture that can then be implemented. Furthermore, the use of English language papers only may have impacted the richness of the data included in this review.

## 6. Conclusions

Medication errors are a common problem that places a massive burden on healthcare systems and are often avoidable by implementing effective preventive strategies. A critical tenant to measure the effectiveness of a reporting system is to measure how effectively the attained information is implemented to enhance patient safety. A successful medication error reporting program has the following characteristics: safe for the reporter, results in useful recommendations and effective changes, includes everyone, and is supported with required resources. Organizations must adopt a successful reporting environment for the medication use process to evolve into a safer practice. It is the responsibility of the organization to provide an environment to its users’ where reporting is conducted in a systematic, ever-evolving manner so that medication is prescribed using a safer infrastructure.

## Figures and Tables

**Table 1:** Characteristics of Successful Reporting Systems.

Non-punitive	No punishment for the reporter as a result of error reporting.
Anonymous	The reporter is not identified by name.
Responsive	Recommendations are disseminated and changes implemented when possible.
Inclusiveness	Engaging everyone (prescriber, pharmacist, nurse, allied health professionals, patient, and family).
Accountability	Holding an individual accountable for continuing unsafe practices.
Supportive environment	Utilize preventive strategies (e.g. information technology) and increase comfort level by considering system design changes.
Summary review	Analyze summary of medication error information on a quarterly, semi-annual, or annual basis.
System-oriented	Focusing on the context and external environment in which an organization operates.
Expert analysis	Understanding the circumstances under which incidents occur and recognizing defects.
Psychological safety	The reporter is able to report without fear of negative consequences of self-image, status, or career.
Resources	Sufficient resources are available where and when they are needed.

## Data Availability

Not applicable.
